# Viruses Broaden the Definition of Life by Genomic Incorporation of Artificial Intelligence and Machine Learning Processes

**DOI:** 10.2174/1570159X20666220420121746

**Published:** 2022-08-31

**Authors:** George B. Stefano, Richard M. Kream

**Affiliations:** 1 Center for Cognitive and Molecular Neuroscience, First Faculty of Medicine, Charles University, Prague, Czech Republic

**Keywords:** Virus, artificial intelligence, long-COVID, mitochondrial genome, eukaryotic genome, SARS-CoV-2, RNA-dependent RNA polymerase

## Abstract

Viruses have been classified as non-living because they require a cellular host to support their replicative processes. Empirical investigations have significantly advanced our understanding of the many strategies employed by viruses to usurp and divert host regulatory and metabolic processes to drive the synthesis and release of infectious particles. The recent emergence of SARS-CoV-2 has permitted us to evaluate and discuss a potentially novel classification of viruses as living entities. The ability of SARS CoV-2 to engender comprehensive regulatory control of integrative cellular processes is strongly suggestive of an inherently dynamic informational registry that is programmatically encoded by linear ssRNA sequences responding to distinct evolutionary constraints. Responses to positive evolutionary constraints have resulted in a single-stranded RNA viral genome that occupies a three-dimensional space defined by conserved base-paring resulting from a complex pattern of both secondary and tertiary structures. Additionally, regulatory control of virus-mediated infectious processes relies on extensive protein-protein interactions that drive conformational matching and shape recognition events to provide a functional link between complementary viral and host nucleic acid and protein domains. We also recognize that the seamless integration of complex replicative processes is highly dependent on the precise temporal matching of complementary nucleotide sequences and their corresponding structural and non-structural viral proteins. Interestingly, the deployment of concerted transcriptional and translational activities within targeted cellular domains may be modeled by artificial intelligence (AI) strategies that are inherently fluid, self-correcting, and adaptive at accommodating temporal changes in host defense mechanisms. An in-depth understanding of multiple self-correcting AI-associated viral processes will most certainly lead to novel therapeutic development platforms, notably the design of efficacious neuropharmacological agents to treat chronic CNS syndromes associated with long-COVID. In summary, it appears that viruses, notably SARS-CoV-2, are very much alive due to acquired genetic advantages that are intimately entrained to existential host processes *via* evolutionarily constrained AI-associated learning paradigms.

## 1 INTRODUCTION

In a highly-cited critical discussion in 2002, Dr. Daniel Koshland formulated a broad-based definition of life based on a set of seven fundamental pillars. These pillars are represented by the appealing anagram known as “PICERAS” [1], which stands for P(rogram), I (mprovisation), C (ompartmentalization), E (nergy), R (egeneration), A (daptability), and S (eclusion). By contrast, traditional scientific thinking has classified viruses as non-living because of their obligate requirement for a cellular host to support their replicative processes. A vast collection of empirical observations has significantly advanced our understanding of the multiple strategies used by viruses to usurp and divert host regulatory and metabolic processes to drive the synthesis and release of infectious particles. The recent emergence of SARS-CoV-2 has revealed the extraordinary diversity of biological mimicry and regulatory control processes that are required for the successful redirection of essential cellular functions. Further elucidation of the complex biochemical and molecular mechanisms that are functionally linked to viral-mediated host cell targeting will require both higher-order empirical observations as well as sophisticated bioinformatics approaches. Accordingly, a redefinition of viruses as living entities with clearly-defined intracellular life cycles is certainly open to critical evaluation and discussion.

The ability of SARS-CoV-2 to initiate and sustain the targeting of diverse cells and cellular processes strongly suggests that it can respond directly to evolutionary constraints. These constraints are highly dynamic and have a direct impact on the information encoded by linear single-stranded (ss) RNA sequences within the ~30kb positive-sense viral genome. Of equal importance, positive evolutionary pressure has programmed the ssRNA SARS CoV-2 genome so that it occupies a distinctive three-dimensional space based on complex patterns of secondary and tertiary structure [2]. The conformational integrity of ss genomic RNA (gRNA) is functionally dependent on the highly conserved base-paired double-stranded (ds)RNA motifs that are uniformly distributed throughout the entire length of the genome. During the early stages of the viral life cycle, the conformational stability of gRNA maintained by the high density of dsRNA may be critically linked to viral-mediated control mechanisms. Among these mechanisms are early translational events that involve the expression of two very large viral polyproteins encoded by 5’ nested open reading frames (ORFs) known as ORF1a and ORF1b [2-5]. The high degree of conformational integrity also confers the gRNA molecules with protection against degradation by endogenous ribonucleases and facilitates efficient transport of nascent transcripts to the endoplasmic reticulum (ER)-associated ribosomes. During later stages of the viral life cycle, seamless integration of virus-mediated synthetic and packaging processes may depend on the precise matching of complementary nucleotide sequences within newly formed full-length gRNA molecules to cognate binding domains within the nucleocapsid (N) proteins. These matches will facilitate the formation of flexible helical ribonucleoprotein/N particles that form the basis of the nascent virions [4, 5]. In summary, the efficacy of these virally-mediated processes may be critically dependent on the functional consequences of complementary conformational matching or shape recognition events that drive high-affinity binding interactions between complementary viral and host nucleic acid and protein domains. Conformational matching is a critical feature of both intracellular and extracellular signaling pathways [6, 7].

Based on convergent lines of evidence discussed above, we contend that temporal deployment of concerted translational and transcriptional activities within targeted cellular domains may be appropriately modeled by artificial intelligence (AI)-associated mechanisms. For example, intracellular entry of infective SARS-CoV-2 gRNA into host cells appears to be synchronized with numerous cellular processes and may be timed specifically to overtake the host cellular machinery and mitochondrial bioenergetics and/or neutralize anti-viral host defense mechanisms [8, 9]. AI-associated models must be inherently flexible, self-correcting, and highly adaptive in their abilities to functionally address a potentially high degree of non-linearity and discontinuity in temporally expressed mosaics of intracellular biochemical and molecular processes. Our brief critical discussion is designed to provide focused critical discussion with representative examples of potentially meaningful lines of evidence curated from selected historical or current SARS/COVID-19 literature from the functional perspective of an evolutionarily fashioned AI-driven viral lifecycle. A working AI model of viral evolution will include selection processes that are functionally beneficial to the virus involving simulations of successive series of machine learning trials. Importantly, the model must reflect the integration of complex replicative processes *via* efficient conformational matching of complementary nucleotide sequences and their corresponding translation products [6, 7], including both structural and non-structural viral proteins. In summary, convergent lines of evidence strongly suggest that viruses are very much alive due to evolutionarily driven genetic advantages that are intimately entrained to attain extensive biological mimicry of existential host processes underlying regulatory control of cellular bioenergetics *via* established AI learning paradigms.

## 2 FOCAL POINTS FOR DISCUSSION AND HYPOTHESIS DEVELOPMENT

### 2.1 Structural Basis of SARS-CoV-2 Gene Expression

**Fig. (1):**
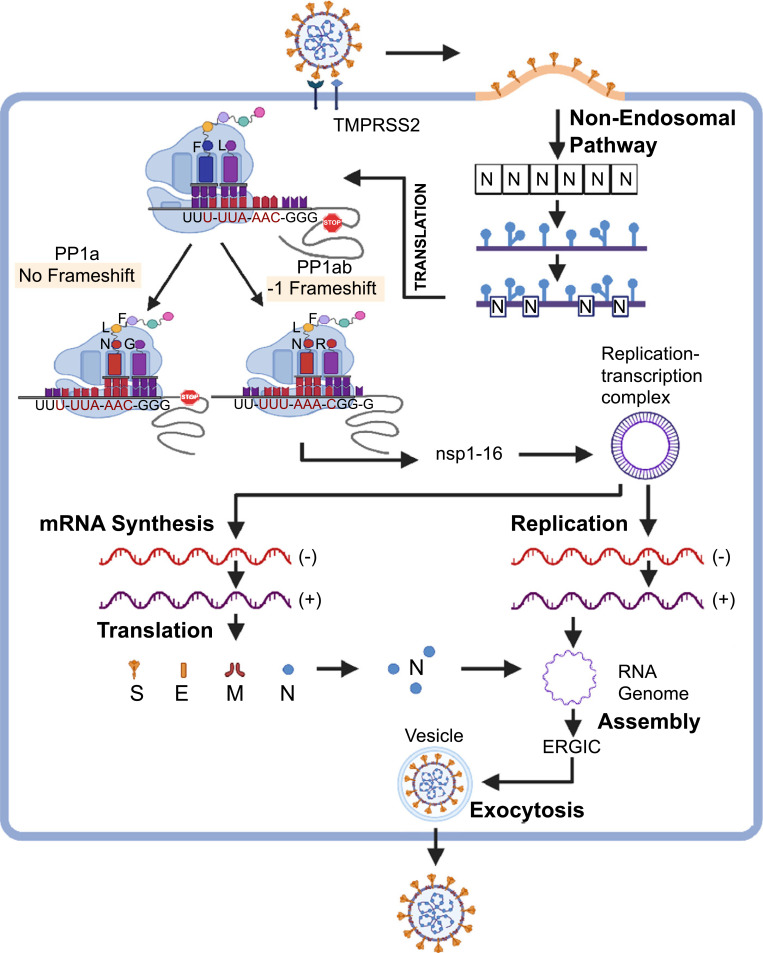
**Schematic representation of the SARS-CoV-2 life cycle from the perspective of AI-associated processes.** Facilitated entry of flexible helical nucleocapsid particles composed of ss gRNA molecules encased in polymerized viral N protein monomers is accomplished *via* a non-endocytotic/non-endosomal-mediated mechanism. As noted in the text, the dimensionality of the data sets to be analyzed would undergo significant expansion when evaluating at least three forms of intracellular gRNA, including (1) intact nucleocapsid particles (represented by linked N-containing boxes, above right), (2) fully denuded particles (represented by a short stem-looped RNA structure), and (3) partially denuded particles (represented by a mosaic of N-containing boxes within a short stem-looped RNA structure). Accordingly, temporally-defined combinations of these defined structures are predicted to confer gRNA molecules with varying degrees of protection against degradation by endogenous ribonucleases that are functionally linked and undergo regulated transport to ribosomes *via* diffusional or cellular scaffold-mediated mechanisms. Early translational events that regulate the expression of viral polyproteins pp1a and pp1ab encoded by 5’ nested ORF1a and ORF1b are regulated by complex conformational processes. Multiple conformational states of the PRF pseudoknot [2, 11, 12] strongly suggest that the intrinsic conformational flexibility of stem-loop 2 (SL2) contained within the PRF domain may have profound consequences with respect to frameshift-mediated efficiency and the calculated ratios of pp1a and pp1ab polyproteins. Accordingly, the differential expression of pp1a versus pp1ab may be operationally linked to intrinsic determinants of PRF efficiency that are functionally dependent on complex conformational changes in SL2, associated ribosomal RNA and protein domains, and gRNA-encoded proteins. Post-translational end proteolytic processing of pp1a and pp1ab is catalyzed by endogenous papain-like and 3C (chymotrypsin)-like domains of nsp3 and nsp5, respectively. These protease domains are internally bonded within the extended amino acid sequence of pp1a, which is a polyprotein that exhibits multiple areas of complex protein folding of underlying secondary and tertiary structural elements [3]. The late phases of the viral life cycle include transcriptional and replicative processes that involve the coordinated recruitment of RTCs. Positive-sense ss gRNA is used as a template to generate both full-length complementary negative-sense ss gRNA intermediates as well as a set of complementary negative-sense subgenomic mRNA (sg-mRNA) molecules. Full-length complementary negative-sense ss RNA genomic intermediates then serve as the templates for the synthesis of positive-sense ss gRNA molecules that associate with the virus N protein to form flexible ribonucleoprotein/nucleocapsid particles. These particles are then packaged into newly formed virions. In addition to the N protein, nested sg-mRNAs encode the conserved structural S, envelope (E), and membrane/matrix (M) proteins that are critically important for virion assembly (Figure modified from [13, 14]).

SARS-CoV and SARS-CoV-2 are beta coronaviruses with high rates of zoonotic transmission. Comparative analyses of these two viral species reveal a high degree of similarity in linear genome construction, sequence homology, corresponding open-reading frames (ORFs), and gene expression profiles [2-5, 10, 11]. Common structural features of the ~30kb positive-sense ss gRNA molecule include a 5’-cap structure, a 3’ poly(A) tail of variable length, and 5′ and 3′ flanking untranslated regions (UTRs) of 265 and 337 nucleotides, respectively. Following receptor-mediated fusion of the viral capsid with the host cell membrane, gRNA and N protein particles appear to enter the target cell *via* a process that is actively coordinated with their transport to ER**-**associated ribosomes; this coordination facilitates early translational events in the viral life cycle (Fig. **1**). Synchronized fusion of viral and cellular host membranes is achieved by high-affinity binding of viral spike (S) protein to the cognate angiotensin-converting enzyme (ACE)-2 receptor/ neutral amino acid transporter SLC6A19 membrane complexes (not shown). This event is coupled with endoproteolytic cleavage of S protein into S1 and S2 subunits *via* the actions of TMPRSS2 (transmembrane protease serine 2). We hypothesize that AI-associated methodologies might be used to model kinetic and stoichiometric advantages gained from the direct and temporally-ordered injection of infectious gRNA molecules into the host cell cytosol during this early phase of infection. The gRNA is then translated into two large nonstructural proteins (NSPs) known as pp1a and pp1ab, with 4405 and 7096 amino acids, respectively. The viral genes that encode these proteins, the 3’ nested ORF1a and ORF1b, respectively, represent 75% of the viral genome. Expression of pp1ab is directed by a -1 programmed ribosomal frameshift (PRF) domain located one nucleotide upstream of the ORF1a termination codon that extends translation through ORF1b. The relative expression of pp1a is 1.5 – 2-fold higher than pp1ab based on a PRF efficiency of 45–70%. This property leads to the functional overexpression of NSPs encoded by ORF1a [11]. Accordingly, the differential expression of pp1a versus pp1ab may be operationally linked to intrinsic determinants of PRF efficiency that are functionally dependent on complex conformational changes in SL2, associated ribosomal RNA and protein domains, and gRNA-encoded proteins. Post-translational proteolytic processing of pp1a and pp1ab catalyzed by endogenous papain-like and 3C (chymotrypsin)-like domains of nsp3 and nsp5, respectively, create 16 multi-functional NSPs that are differentially assembled into complementary interactive replication–transcription complexes (RTCs) (Fig. **1**). We hypothesize that AI-associated algorithms that model conformational protein-protein interactions may be used to elucidate temporally-defined biosynthetic/maturation pathways of the16 multi-functional NSPs that are expressed after multiple cis and potential trans-activated cleavage events targeting pp1a and pp1ab polyproteins. Importantly, conformational matching and shape recognition algorithms can reliably predict spatially-oriented presentations of the catalytically-active sites of nsp3- and/or nsp5 with consensus cleavage sites distributed throughout the linear amino acid sequences of pp1a and pp1ab.

The subsequent phases of the viral life cycle include temporally linked transcriptional and replicative processes that involve coordinated recruitment of RTCs. Nsp12-mediated RNA-dependent RNA polymerase (RdRP) activity maintains the catalytic center, while nsp7 and nsp8 are allosteric modulators within the RdRP holoenzyme complex [4, 11]. The positive-sense ss gRNA is used as a template to generate both full-length complementary negative-sense ss gRNA intermediates as well as a set of complementary negative-sense subgenomic mRNA (sg-mRNA) molecules that are derived from 3’ nested sg-ORFs. Full-length complementary negative-sense ssRNA genomic intermediates then serve as the templates for the synthesis of positive-sense ss gRNA molecules (Fig. **1**). These positive-sense ss gRNA molecules then associate with the virus N protein to form flexible ribonucleoprotein/nucleocapsid particles that are subsequently packaged in newly formed virions. In addition to the N protein, nested sg-mRNAs encode the conserved structural spike (S), envelope (E), and membrane/matrix (M) proteins that are critically important for virion assembly. The nested sg-mRNAs also encode seven accessory proteins (3a, 3b, 6, 7a, 7b, 8, and 9b) with diverse regulatory activities that attenuate the innate immune responses [4, 11].

### 2.2 Potential Applications of AI-Associated Models

In a recent critical discussion paper, Emmert-Streib and colleagues [15] reviewed multiple perspectives on the current status of AI and compared some of the widely- employed methods for machine learning and statistical analysis. In this manuscript, the authors highlighted several points of discussion and concluded that AI is an effective method that employs mathematical learning algorithms that can undergo self-directed adjustment *via* specific learning rules and that the intellectual components of AI reside in the software-based implementation of these learning rules. Current successful applications of AI are those that address relatively simple questions *via* in-depth analyses of complex high-dimensional data sets. Accordingly, AI approaches include those currently employed in machine learning paradigms and statistical methodologies. Within this functional context, our hypothesis that the temporal deployment of concerted viral-mediated translational and transcriptional activities within targeted cellular domains may be appropriately modeled by AI-associated methods appears to be feasible and amenable to testing *via* analysis of large, high-dimensional data sets. The underlying rationale for adopting AI approaches may reside in the limitations inherent in any attempt to single-handedly evaluate the exhaustive and sometimes conflicting data sets that attribute multiple functional regulatory roles and potential catalytic activities to the majority of viral NSPs, structural proteins, and accessory proteins [2-5, 11]. There is currently a substantial amount of detailed information pertaining to the functional activities of newly-expressed viral proteins and their role in promoting concerted translational and transcriptional activities at all stages of the viral life cycle. Large data sets have been compiled from controlled studies *via* complementary biochemical, molecular, and structural biological methods, as well as *in vitro* and *in silico* methods and model systems. Many of these compilations were designed to collect dependent measures from data sets at a steady state without the kinetic parameters that are necessary to model temporal changes in viral-host interactions *in vivo*.

### 2.3 Evolutionary-Driven Complexity in the SARS-CoV-2 Transcriptome: Conformational Matching with Secondary and Tertiary Structural Elements of gRNA

Huston and colleagues [2] reported the results of an important complementary study that utilized high-resolution structure probing methodologies (SHAPE-MaP) to document the complete secondary structure of full-length SARS-CoV-2 gRNA under simulated *in vivo* conditions. The authors utilized a very similar experimental design to that described above [4] and established that SARS-CoV-2 gRNA occupies a 3-dimensional space based on complex patterns of the secondary and tertiary structure under simulated *in vivo* conditions. The results of this study strongly suggest that functional coupling of conformationally-matched RNA and protein domains is intimately involved in the regulatory control of transcriptional and translational events within the viral life cycle. The conformational integrity of gRNA molecules is maintained by highly conserved base-pairing sequences in folded dsRNA motifs that are uniformly distributed at high frequencies throughout the entire length of the viral genome. Interestingly, SARS-CoV-2 gRNA displays a shorter median base-pairing distance compared to other positive-sense viral gRNAs. Among the possible interpretations of this finding, spatial compaction of high-density dsRNA motifs confers protection against degradation by endogenous ribonucleases while simultaneously facilitating N protein-associated nucleocapsid formation. Interestingly, current structural predictions based on conformational states of the well-studied PRF pseudoknot [2, 11, 12] strongly suggest that the intrinsic conformational flexibility of stem-loop 2 (SL2) contained within the PRF domain may have profound functional consequences with respect to the frameshift-mediated regulation of pp1a and pp1ab polyproteins encoded by ORF1a and ORF1b [2]. The authors suggested that this property may endow SL2 with “on-off” biological switching properties that are functionally associated with both its native conformation (that permits some frameshifting) and its unfolded state, in which extensive base pairing occurs with complementary structures outside the PRF domain (and thus permits almost no frameshifting). Accordingly, the differential expression of pp1a *versus* pp1ab may be operationally linked to intrinsic determinants of PRF efficiency that are functionally dependent on complex conformational changes in SL2. Finally, the rate of ribosomal translocation may be effectively reduced by conserved, highly-folded regions of dsRNA located at the boundaries of ORF1a and ORF1b to optimize post-translational proteolytic processing events and conformational maturation of NSPs that are derived from newly translated pp1a and pp1ab polyproteins. To summarize, complex transcriptional and translational processes that drive the successful completion of the viral life cycle appear to be exquisitely regulated by conformational matching of interactive RNA and protein domains that are encoded by information maintained within evolutionarily-responsive linear sequences of viral gRNAs.

## CONCLUSION AND FUTURE DIRECTIONS

In this brief review, we identify and discuss major themes and new evidence supporting the contention that viruses are very much alive. Specifically, we discuss the fact that SARS-CoV-2 can respond and incorporate evolutionarily-driven genetic advantages that permit them to overtake numerous essential host cell processes. Furthermore, the efficacy of virus-mediated mimicry of these cellular events may be directly dependent on conformational matching processes that drive high affinity complementary binding interactions of viral and host nucleic acid and protein domains. We surmise that virus-mediated temporal translational and transcriptional activities within targeted cellular domains may be appropriately modeled by AI-associated methods that are inherently flexible, self-correcting, and highly adaptive in their capacity to evaluate datasets with substantial non-linearity and discontinuity. Taken together, through 3.5 billion years of evolutionarily-directed complementary conformational matching events using the same genetic building blocks, viruses, bacteria, and eukaryotic cells emerge with properties that are optimized for survival. We hypothesize that this process is designed to accept and utilize change to survive and advance in response to changing environmental and natural selection processes that are occurring simultaneously. Thus, “change” mechanisms are built into life processes and occur naturally (*e.g.*, mutations in RNA polymerase that facilitate resistance to protease-mediated degradation). In this regard, internal intrinsic shape recognition processes are subjected to further selection based on resulting changes in shape that are governed largely by natural selection and kinetic-dependent random selection. The frequency of intrinsic changes/mutations incorporated into the respective genomes decreases as the cell becomes more complex and involved in multi-cellular events.

During the past few years, the International Committee on Taxonomy of Viruses (ICTV) has extended viral taxonomic criteria to include evolutionary relationships that provide functional links between distantly related viruses [16, 17]. Accordingly, the ICTV has adopted a 15-rank classification hierarchy that is closely aligned with the Linnaean taxonomic system to accommodate an expanded spectrum of genetic divergence within the virosphere. This newly-expanded taxonomic classification system certainly supports our major hypothesis that viruses are both obligate intracellular parasites as well as living entities that are sustained by complementary matching and conformational recognition with respect to both their intrinsic processes as well as those involved in their survival and interactions with their targeted hosts.

As discussed in the earlier sections, a working AI model of virus evolution should include functionally beneficial selection processes that simulate those that confer mutational advantages in the variant species. As suggested by Ho and colleagues [18], these responses rely on the role of overprinting or overlapping genes. Interestingly, 53% of virus genes are overlapping or overprinted (compared to only 1% of the mammalian genome). Thus, overprinting is a common phenomenon in viral species that reinforces our hypothesis concerning viral influences that drive evolution in both prokaryotes and eukaryotes. Of note, this phenomenon increases the probability that viruses will be capable of incorporating mutations that modify host cell processes [18]. Hence, viral complexity emerges from an ever-evolving source of mutational change that continuously fine-tunes itself to maintain compatibility with host processes that are vital for its survival; in some circumstances, these changes may also inadvertently benefit the host. Taken together, this dynamic bidirectional communication has the potential to share genetic information with other prokaryotic and eukaryotic genomes. Thus, we conclude that viruses are alive and are supported by complex genetic processes that facilitate ongoing improvements in their reproductive and metabolic capacities as a basis of their unique survival strategies. An improved understanding of this complex life form will promote the development of therapeutic options for various virally-mediated and host-associated disorders.

## LIST OF ABBREVIATIONS

ACEs: Angiotensin-converting Enzymes
AI: Artificial Intelligence
CNS: Central Nervous System
COVID: Coronavirus Disease
3C: 3-chymotrypsin
dsRNA: Double-stranded Ribonucleic Acid
E protein: Envelope protein
ER: Endoplasmic Reticulum
gRNA: Genomic Ribonucleic Acid
ICTV: International Committee on Taxonomy of Viruses
M protein: Membrane/Matrix Protein
N protein: Nucleocapsid Protein
NSP: Non-structural Protein
ORFs: Open Reading Frames
pp1a/b: Polyproteins 1a/b
PRF: Programmed Ribosomal Frameshift
RdRP: Ribonucleic Acid Dependent Ribonucleic Acid Polymerase
RTC: Replication Transcription Complex
SARS-CoV: Severe Acute Respiratory Syndrome
sg-mRNA: Subgenomic Messenger Ribonucleic Acid
SHAPE-MaP: Selective 2'-hydroxyl Acylation Analyzed by Primer Extension and Mutational Profiling
SLC6A19: Solute Carrier Family 6 Member 19
SL2: Stem-loop 2
S protein: Spike Protein
ss-gRNA: Single-stranded Genomic Ribonucleic Acid
ssRNA: Single-stranded Ribonucleic Acid
TMPRSS2: Transmembrane Protease Serine 2
UTR: Untranslated Region
